# Complication of Trigger Point Injections: Retained Injection Needle Causing Pneumothorax

**DOI:** 10.7759/cureus.95679

**Published:** 2025-10-29

**Authors:** Rachel Reindorf, Jonathan Saju, Seung J Lee, Kanchana Gattu, Lynn G Stansbury, Thelma Wright

**Affiliations:** 1 Anesthesiology, University of Maryland Medical Center, Baltimore, USA; 2 Anesthesiology, Campbell University School of Osteopathic Medicine, Lillington, USA; 3 Laboratory Medicine and Pathology, University of Washington School of Medicine, Seattle, USA

**Keywords:** chronic low back pain (clbp), myofascial pain syndrome, needle breakage, pneumothorax (ptx), trigger point injection, ultrasound guidance

## Abstract

Trigger point injections (TPIs) are widely used for myofascial pain syndrome and are generally safe, though rare complications, such as pneumothorax, have been reported. We present a rare case of a retained injection needle causing pneumothorax following TPI. A 38-year-old male with chronic back pain, chronic obstructive pulmonary disease (COPD), and prior spinal surgery developed right-sided pleuritic pain and dyspnea several hours after TPI. Imaging revealed a right apical pneumothorax and a retained metallic needle within the thoracic cavity. The needle was successfully removed via video-assisted thoracoscopic surgery (VATS), and the patient was discharged the same day without complications. This case highlights the importance of procedural vigilance during TPIs, including careful documentation, appropriate needle selection based on anatomy, and post-procedure inspection of instruments. The use of ultrasound guidance may further reduce risks such as pleural puncture and needle retention. Standardized safety measures are essential to prevent such serious, avoidable complications in routine pain management practice.

## Introduction

Myofascial pain syndrome is the most common cause of disabling chronic back pain, present in up to 90% of cases [1-3]. The primary treatment of myofascial pain syndrome is trigger point injections (TPIs). These involve percutaneous injection of local anesthetic or a mixture of local anesthetic and steroids into palpably tight bands of affected skeletal muscle [4-6]. Although generally considered low-risk, TPIs have been associated with rare complications such as intramuscular hematoma, vasovagal syncope, iatrogenic spinal cord injury, and pneumothorax [7-9]. We present a rare complication of TPI in which an injection needle used for the procedure was retained in the thoracic cavity, resulting in pneumothorax. The present case is unique in that the pneumothorax resulted from the retention of a needle fragment, rather than a transient pleural puncture, representing a persistent source of pleural irritation, infection risk, and potential migration, necessitating surgical intervention. This case emphasizes the need for improved procedural safeguards to uphold patient safety and standardization regarding needle selection based on anatomy and injection depth.

## Case presentation

A 38-year-old male with a BMI of 23 and a history of chronic low back pain, prior L5-S1 fusion, migraines, obstructive sleep apnea (OSA), chronic obstructive pulmonary disease (COPD), Factor V Leiden mutation (not on anticoagulation), and active tobacco use, presented to our emergency department with right-sided posterior back and lung pain. The patient reported that he had undergone TPIs earlier that day for chronic neck and low back pain at an outside pain clinic. Upon procurement of the patient’s medical records from the outside pain clinic, there was no mention of needle length/gauge or whether ultrasound guidance was used. There was also no mention of whether dry needling or injectates were used, as well as what the specific muscles or landmarks injected into were. The patient reported that 2.5 hours after the TPI, he developed right-sided back pain that worsened with inspiration. He took baclofen, ibuprofen, and rimegepant, with some relief, but subsequently experienced fever and chills. The pain progressed to a stabbing quality, prompting him to contact the outside pain clinic. He was advised by the RN on call that he should report to emergency care for evaluation, including a chest X-ray.

**Figure 1:**
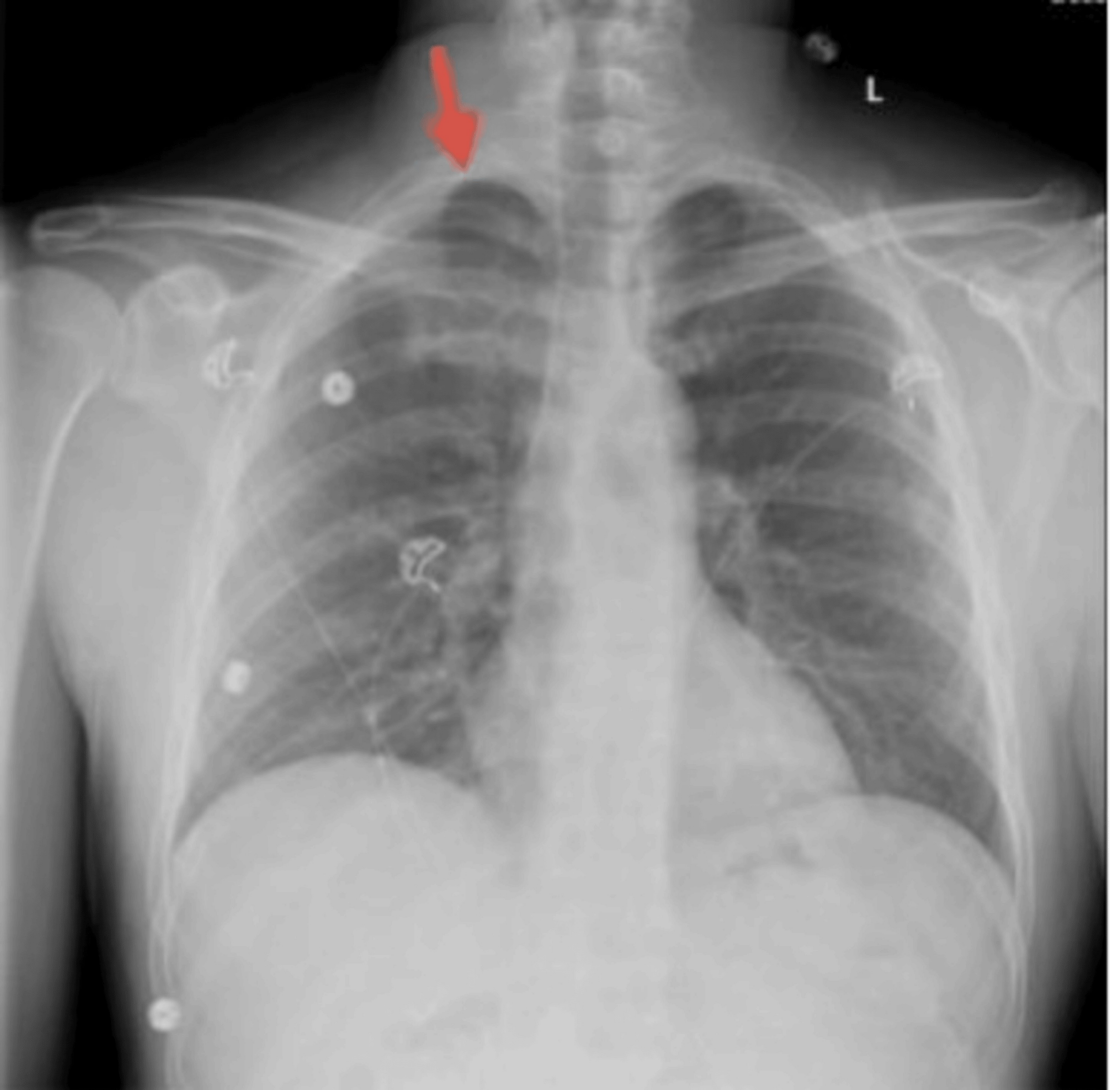
Chest X-ray with arrow pointing to the R apical pneumothorax (posteroanterior view)

**Figure 2:**
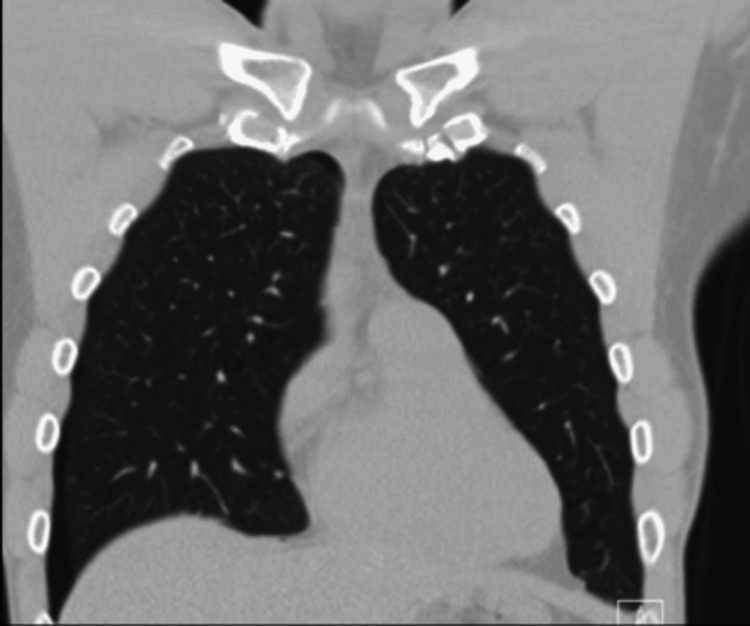
CT scan showing right apical and anterior pneumathoraxes (coronal view)

**Figure 3:**
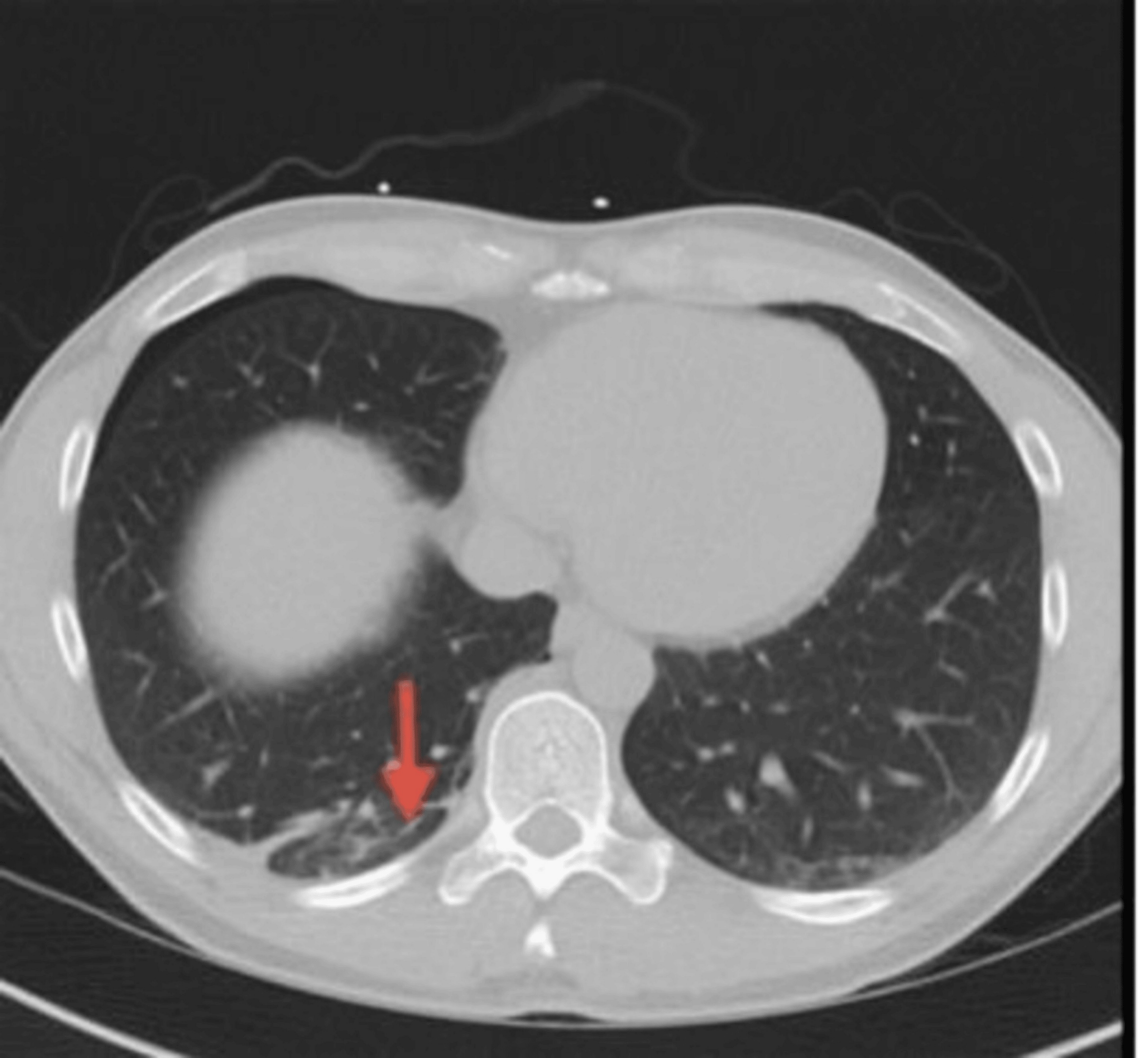
CT scan with arrow pointing to the retained injection needle (axial view)

**Figure 4:**
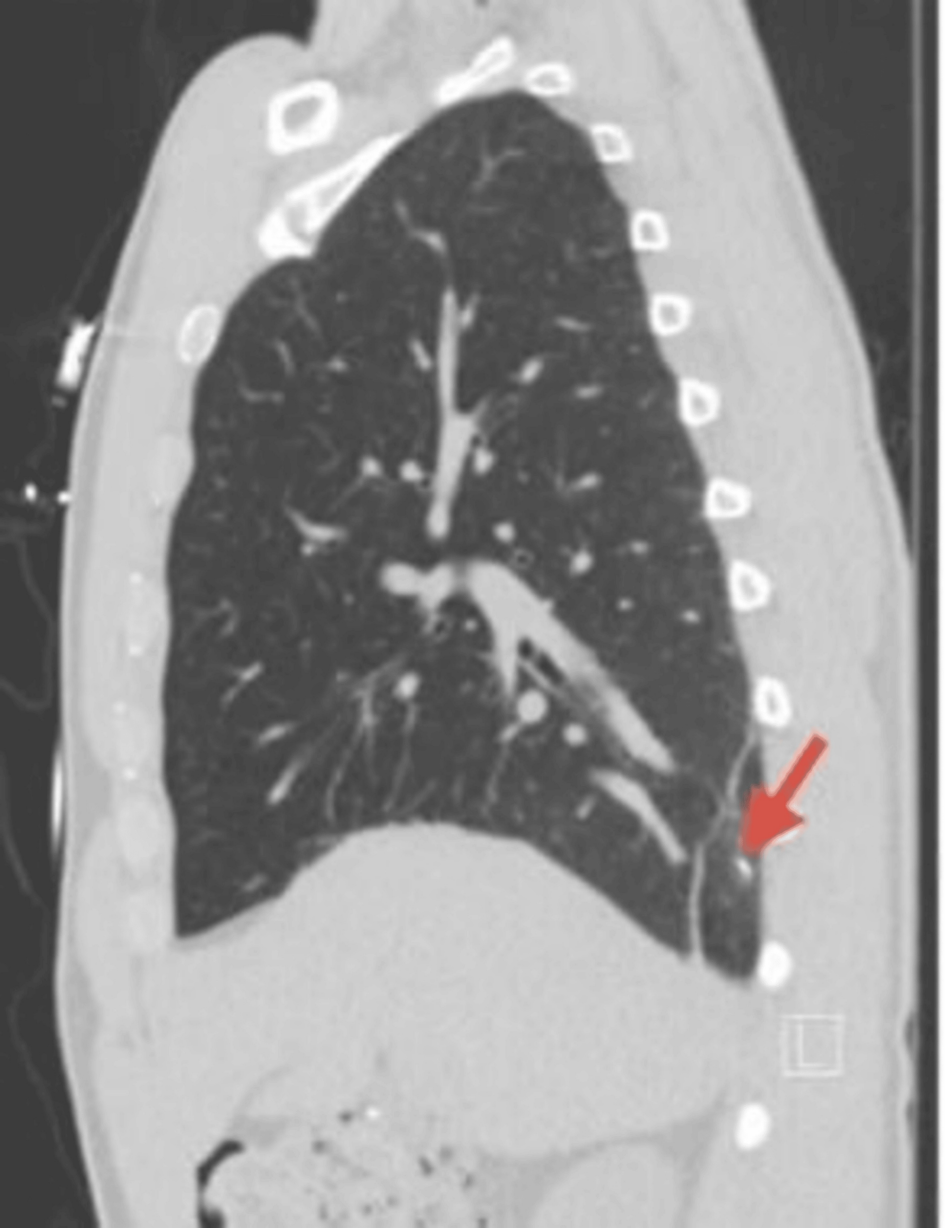
CT scan with an arrow pointing to the retained injection needle (sagittal view)

Upon presentation to the ED, the patient continued to experience pain along with the new onset of shortness of breath. Laboratory findings were significant for a WBC count of 11.2, hemoglobin of 15.7, and platelets of 299. A chest X-ray revealed a pneumothorax as well as a foreign metallic object (Figure 1). A subsequent computed tomography (CT) scan confirmed the presence of apical and anterior pneumothoraxes with the needle embedded in the parietal pleura (Figures 2-4). The patient was referred to thoracic surgery for evaluation for video-assisted thoracoscopic surgery (VATS) for the removal of the retained needle.

Intraoperative analgesia and postoperative care 

**Figure 5:**
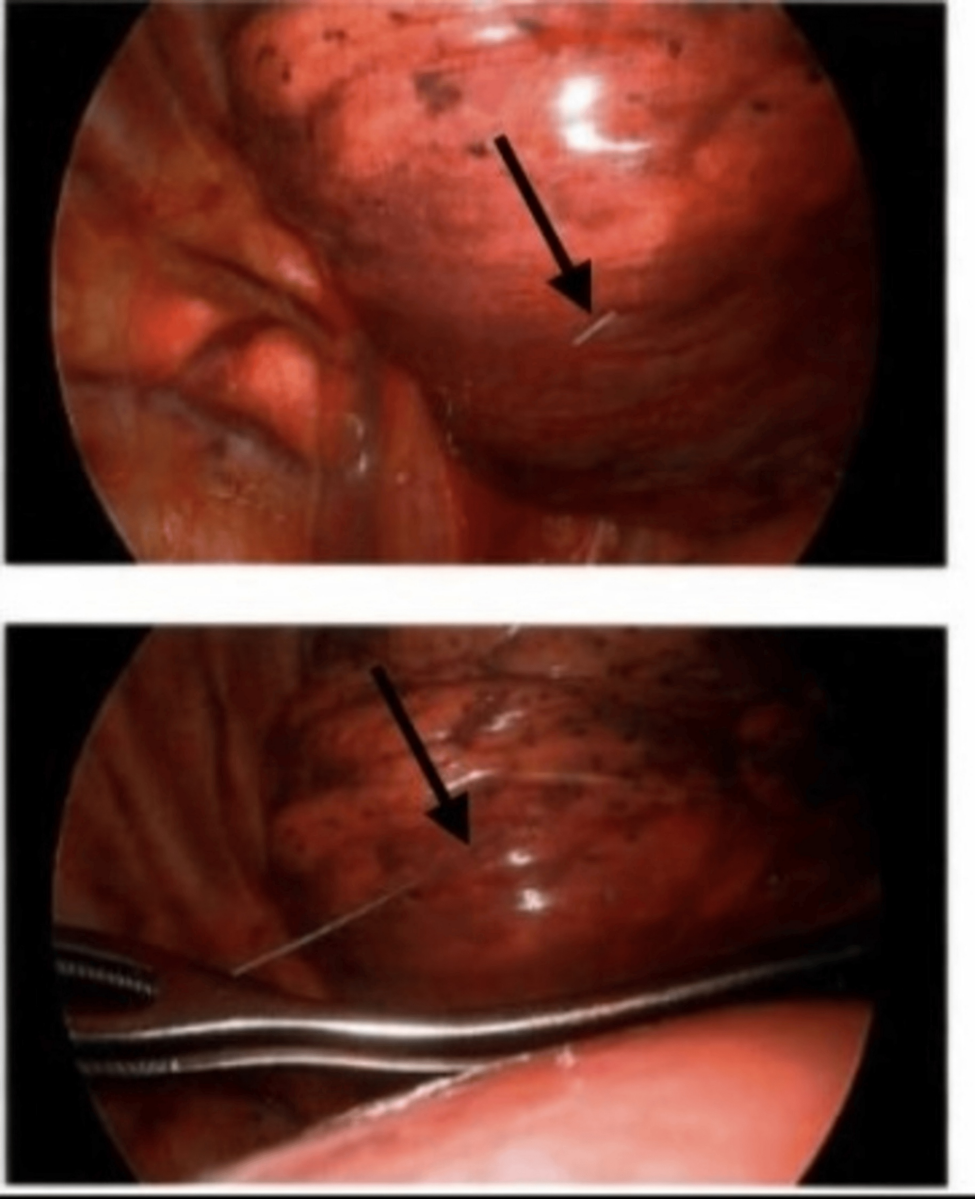
Intraoperative removal of the retained injection needle

VATS was performed under general anesthesia with lung isolation, which was well-tolerated. The retained needle was successfully visualized and removed, as shown in Figure 5. Preoperatively, the patient received 1 gram of acetaminophen, and intraoperatively, he was given 20 mg of IV methadone for analgesia because of his chronic use of oxycodone 15 mg every four hours. Additionally, 40 cc of 0.25% bupivacaine was injected into the incision sites by the surgical team for local analgesia. The patient received 30 mcg of IV dexmedetomidine hydrochloride at the end of the case in the OR and an additional 20 mcg in the recovery area for continued pain management. The patient experienced no complications and was discharged home the same day.

## Discussion

TPIs are often considered low-risk procedures with minimal complications. However, there is a known, though rare, risk of pneumothorax, particularly when injections are performed in the cervicothoracic region [10]. This risk of pneumothorax was amplified by the patient’s history of COPD and chronic tobacco use (pack per day since 1998), both of which contribute to the development of pneumothorax [11]. The patient also had significant environmental exposure to air pollutants during his 12-year service in the military when he was exposed to burn pits in Kuwait, Iraq, and Egypt, which further exacerbated the risk of developing pneumothorax [12]. These procedures are typically done in outpatient clinic settings, with patients discharged shortly afterward and minimal post-procedure monitoring. This underscores the importance of providing patients with thorough discharge and return instructions.

Currently, there is limited guidance regarding the appropriate needle size for TPIs, especially concerning variations in patient body habitus. The retained 1.5-inch needle fragment indicates that a 2.5- or 3-inch needle was likely used. Injections in the cervicothoracic region carry a high risk of pleural puncture, and the use of such long needles is strongly discouraged unless performed under real-time image guidance. This raises concerns about the lack of standardization in needle selection for these procedures. The current best practices for needle selection based on anatomy and injection depth is as follows: for most injections, a 27-gauge, 1.25-inch needle is used; for deeper injections, a 25-gauge, 2.5-inch needle is used; and for very deep injections, a 22-gauge, 3-inch needle is used. When considering the selection of needle size, it is important to use the smallest needle consistent with the provider’s ability to reach the trigger point and cause the least pain to the patient [13].

Emerging literature supports the use of ultrasound guidance during TPIs to reduce the risk of direct pleural puncture during the procedure. While ultrasound may increase procedure and billing time, the potential to reduce serious complications is an important consideration [14]. Most importantly, careful visual inspection must be undertaken post-procedure to recognize potential complications like needle retention in this case.

## Conclusions

While TPIs remain a widely used and generally safe intervention for myofascial pain syndrome, this case highlights the potential for serious complications, including pneumothorax and retained injection needles. The incident underscores the critical need for standardized guidelines on needle selection based on patient anatomy and postoperative observation for needle retention and other complications. We strongly advocate for the routine use of ultrasound guidance for injections in high-risk anatomical regions. As these procedures continue to be administered in outpatient settings with minimal monitoring, clear procedural instructions and vigilant follow-up are essential. Incorporating these measures into clinical practice could significantly reduce adverse outcomes and improve overall patient care in the management of chronic back pain.

## References

[1] Ammer K, Ebenbichler G, Bochdansky T (2022). Low back pain—a disease or condition of impaired functional health? Definition-inherent consequences for the comprehensive care of back pain patients. BioMed.

[2] Chen CK, Nizar AJ (2011). Myofascial pain syndrome in chronic back pain patients. Korean J Pain.

[3] Coelho DM, Barbosa RI, Pavan AM, Oliveira AS de, Bevilaqua-Grossi D, Defino HLA (2014). Prevalence of myofascial dysfunction in patients with low back pain [Article in Portuguese]. Acta Fisiátrica.

[4] Dua A, Chang KV (2025). Myofascial pain syndrome. StatPearls [Internet].

[5] Hammi C, Schroeder JD, Yeung B (2025). Trigger point injection. StatPearls [Internet].

[6] Hamzoian H, Zograbyan V (2023). Trigger point injections versus medical management for acute myofascial pain: a systematic review and meta-analysis. Cureus.

[7] Zheng J, Song H, Cai S (2017). Evaluation of clinical significance and risk factors of incidental parathyroidectomy due to thyroidectomy. A single-center retrospective clinical study. Medicine (Baltimore).

[8] Kim JB, Chang MC (2021). Spinal cord injury by direct damage during trigger point injection: a case report. J Int Med Res.

[9] Morjaria JB, Lakshminarayana UB, Liu-Shiu-Cheong P, Kastelik JA (2014). Pneumothorax: a tale of pain or spontaneity. Ther Adv Chronic Dis.

[10] Climenhage BP, DO NYL, MD LMV, MD CWH (2025). Bilateral pneumothoraces after trigger point injection therapy: a case report. FCEP. https://fcep.org/bilateral-pneumothoraces-after-trigger-point-injection-therapy-a-case-report/.

[11] Hobbs BD, Foreman MG, Bowler R (2014). Pneumothorax risk factors in smokers with and without chronic obstructive pulmonary disease. Ann Am Thorac Soc.

[12] Liu YW, Kao CN, Ho CC, Chou SH, Chen PC, Huang SH (2025). Effect of environmental factors on postoperative recurrent primary spontaneous pneumothorax: a case-crossover study. Respir Res.

[13] Filner BE (2008). Tips on practicing good technique for inactivating trigger points while avoiding pitfalls and minimizing complications. Pract PAIN Manag. Published online December.

[14] Botwin KP, Sharma K, Saliba R, Patel BC (2008). Ultrasound-guided trigger point injections in the cervicothoracic musculature: a new and unreported technique. Pain Physician.

